# Self-connected CuO–ZnO radial core–shell heterojunction nanowire arrays grown on interdigitated electrodes for visible-light photodetectors

**DOI:** 10.1038/s41598-022-10879-5

**Published:** 2022-04-27

**Authors:** Andreea Costas, Camelia Florica, Nicoleta Preda, Cristina Besleaga, Andrei Kuncser, Ionut Enculescu

**Affiliations:** National Institute of Materials Physics, Nanostructures Laboratory, 405A Atomistilor Street, 077125 Magurele, Ilfov Romania

**Keywords:** Nanowires, Nanophotonics and plasmonics

## Abstract

An original photodetector system based on self-connected CuO–ZnO radial core–shell heterojunction nanowire arrays grown on metallic interdigitated electrodes, operating as visible-light photodetector was developed by combining simple preparation approaches. Metallic interdigitated electrodes were fabricated on Si/SiO_2_ substrates using a conventional photolithography process. Subsequently, a Cu layer was electrodeposited on top of the metallic interdigitated electrodes. The CuO nanowire arrays (core) were obtained by thermal oxidation in air of the Cu layer. Afterwards, a ZnO thin film (shell) was deposited by RF magnetron sputtering covering the surface of the CuO nanowires. The morphological, structural, compositional, optical, electrical and photoelectrical properties of the CuO nanowire arrays and CuO–ZnO core–shell nanowire arrays grown on metallic interdigitated electrodes were investigated. The performances of the devices were evaluated by assessing the figures of merit of the photodetectors based on self-connected CuO–ZnO core–shell heterojunction nanowire arrays grown on the metallic interdigitated electrodes. The radial p–n heterojunction formed between CuO and ZnO generates a type II band alignment that favors an efficient charge separation of photogenerated electron–hole pairs at the CuO–ZnO interface, suppressing their recombination and consequently enhancing the photoresponse and the photoresponsivity of the photodetectors. The electrical connections in the fabricated photodetector devices are made without any additional complex and time-consuming lithographic step through a self-connecting approach for CuO–ZnO core–shell heterojunction nanowire arrays grown directly onto the Ti/Pt metallic interdigitated electrodes. Therefore, the present study provides an accessible path for employing low dimensional complex structures in functional optoelectronic devices such as photodetectors.

## Introduction

An important characteristic of scientific and technical evolution is related to packing more functionalities in devices. Consequently, nanomaterials and nanotechnology become key elements in the development of many areas including materials science, electronics, computer science, energy, environment or life sciences. Electronic and optoelectronic devices such as field effect transistors, light emitting diodes, photodetectors or solar cells are faster, smaller, energy efficient and cost-effective using nanostructures or nanostructured materials as building blocks^1–6^. Visible-light photodetectors are an important class of optoelectronic devices, finding applications in communication, wearable devices, biological imaging, environmental research, molecular sensing, etc.^7–9^.

Nowadays, the rapid development and implementation of Internet of Things (IoT) using new and advanced technologies have inspired researchers to develop visible-light photodetectors based on one dimensional (1D) nanomaterials^8,9^. Nanowires represent a distinctive category of 1D nanostructures, thoroughly investigated due to their highly anisotropic shape (diameters of 1–100 nm and lengths up to tens of micrometers), being suitable for applications in different areas such as optoelectronics, spintronics, photocatalisys, sensing, biotechnology, etc.^10–13^. Semiconductor nanowires have been widely explored as novel building blocks for advanced miniaturized optoelectronic devices, easily finding applications in field effect transistors^14,15^, solar cells^16^, light emitting diodes^17^, photodetectors^18,19^, photoelectrochemical water splitting^20^, etc. Different from bulk materials and thin films, owed to their high surface-to-volume ratio and large specific surface area, semiconductor nanowires are expected to enhance light confinement and photosensitivity in photodetectors^21^. However, one main challenge in semiconductor nanowires is related to the impact of the high density of surface states on their electrical properties as a consequence to the quantum-confined geometry. These point defects may act as nonradiative carrier traps that can decrease the electrical conductivity and limit the nanowire based photodetector performances^22^. Recently, two components based heterostructures have stimulated tremendous research efforts due to their potential use in obtaining improved optoelectronic devices compared to their single material counterparts^23^. A successful path to improve and fine tune the optoelectronic performances of the nanowires is to develop radial core–shell heterojunction nanowires which combine the features of two different semiconductor materials in order to enhance their individual properties, the core–shell architecture leading to advanced band engineering capability^24^. Hence, in the last years, core–shell heterojunction nanowires based on metal oxides emerged as promising new materials capable of encoding new and advanced functionalities that are essential for the next generation miniaturized nanowire based photodetectors^25,26^. Accordingly, various core–shell heterojunctions were used in order to fabricate photodetectors based on single (ZnO–Cu_x_O^27^, CuO–ZnO^28^, ZnO/ZnS^29^, ZnO/Ws_2_^30^, ZnO/AlN^31^, etc.) or on arrays (ZnO–Cu_2_O^32^, ZnO/CuCrO_2_^33^, ZnO/NiO^34^, ZnO–Co_3_O_4_^35^, ZnO/SnO_2_^36^, ZnO/Ga_2_O_3_^37^, ZnO/Si^38^, etc.) of radial core–shell nanowires. In spite of considerable efforts and progress made by researchers in fabricating semiconductor nanowire based photodetectors, there are still challenges that needs to be overcome, mostly related to material properties tuning and device architecture engineering, leaving room for new research.

Some metal oxides are low cost and non-toxic semiconductor materials that can be easily prepared by different pathways. The bandgap of such metal oxides can be tuned during their synthesis allowing tailored light absorption from the UV up to IR spectral domains, thus making them suitable for applications as photodetectors^32^. Amidst metal oxides, CuO is a p-type semiconductor with specific parameters including a narrow band gap (1.2–2 eV) and a high optical absorption in the visible range^39^. ZnO is a n-type semiconductor with a wide direct band gap (3.37 eV), large exciton binding energy (60 meV) at room temperature, and high electron mobility (up to 200 cm^2^ V^−1^ s^−1^)^14^. By joining CuO and ZnO into a radial core–shell nanowire configuration, a type II heterojunction is formed, leading to a structure suitable for advanced photodetectors with broad detection range^27,28,40^. The staggered gap type II heterojunction promotes an efficient charge separation of photogenerated electron–hole pairs along the CuO–ZnO interface, generating a built-in electric field and consequently enhancing the light absorption efficiency^27,28,40^. Until now, only a few approaches were used to obtain CuO–ZnO core–shell heterojunction nanowire arrays, the cores consisting in CuO nanowires and the shell being a ZnO thin film entirely covering the surface of the CuO nanowires^28,41–43^. Thus, the CuO nanowires (core) were prepared by thermal oxidation in air^28,41–44^, while the ZnO thin film shell was obtained by radio-frequency (RF) magnetron sputtering^28^, thermal decomposition^41–43^ or atomic layer deposition^44^. Moreover, we recently fabricated p–n radial heterojunction diodes based on single CuO–ZnO core–shell nanowires behaving as photodetectors^28^.

Metallic interdigitated electrodes are often employed as a platform for detecting devices due to their low Ohmic drop, increased signal to noise ratio and fast detection speed given by their alternating “finger-like” architecture with gaps typically in the micrometer range^45,46^.

In the current study, we aim to develop a new type of photodetector system based on self-connected CuO–ZnO core–shell heterojunction nanowire arrays grown on metallic interdigitated electrodes, in order to explore their potential application in visible-light photodetectors. The nanowire based photodetector system was developed using the following preparation steps: (i) metallic interdigitated electrodes were fabricated on Si/SiO_2_ substrates using a conventional photolithography process; (ii) a Cu layer was electrodeposited on top of the metallic interdigitated electrodes; (iii) CuO nanowire arrays (core) were prepared by thermal oxidation in air of the Cu layer; and (iv) ZnO thin film shell was deposited by RF magnetron sputtering, covering the surface of the CuO nanowires. The morphological, structural, compositional and optical properties of the CuO nanowire arrays and CuO–ZnO core–shell nanowire arrays grown on metallic interdigitated electrodes were evaluated. Afterwards, the electrical and photoresponse properties of the photodetector devices were assessed in dark and under illumination. The figures of merit of the fabricated photodetectors were estimated, their performances being compared with state-of-the-art core–shell nanowire arrays based photodetectors. The reported devices have a major advantage: the electrical connections in the fabricated photodetectors are obtained without any additional complex and time-consuming lithographic steps due to the self-connected CuO–ZnO core–shell heterojunction nanowire arrays grown directly onto the Ti/Pt metallic interdigitated electrodes. The current work represents a step-forward on the research for next generation miniaturized visible-light photodetectors based on core–shell heterojunction nanowire arrays.

## Materials and methods

The chemical reagents used in the copper electrodeposition (Cu_2_SO_4_·5H_2_O and H_2_SO_4_) were bought from Merck and used as received without further purification. Deionized water was provided by a Millipore water purification system. The n-type doped Si/SiO_2_ wafers having a diameter of 3 inch were acquired from Si-Mat Silicon Materials. The titanium (99.995% purity), platinum (99.99% purity) and zinc oxide (99.999% purity) sputtering targets having a diameter of 2 inch were purchased from Kurt J. Lesker Company Ltd. (UK).

### Fabrication of Ti/Pt/Cu metallic interdigitated electrodes on Si/SiO_2_ substrates

**Figure 1:**
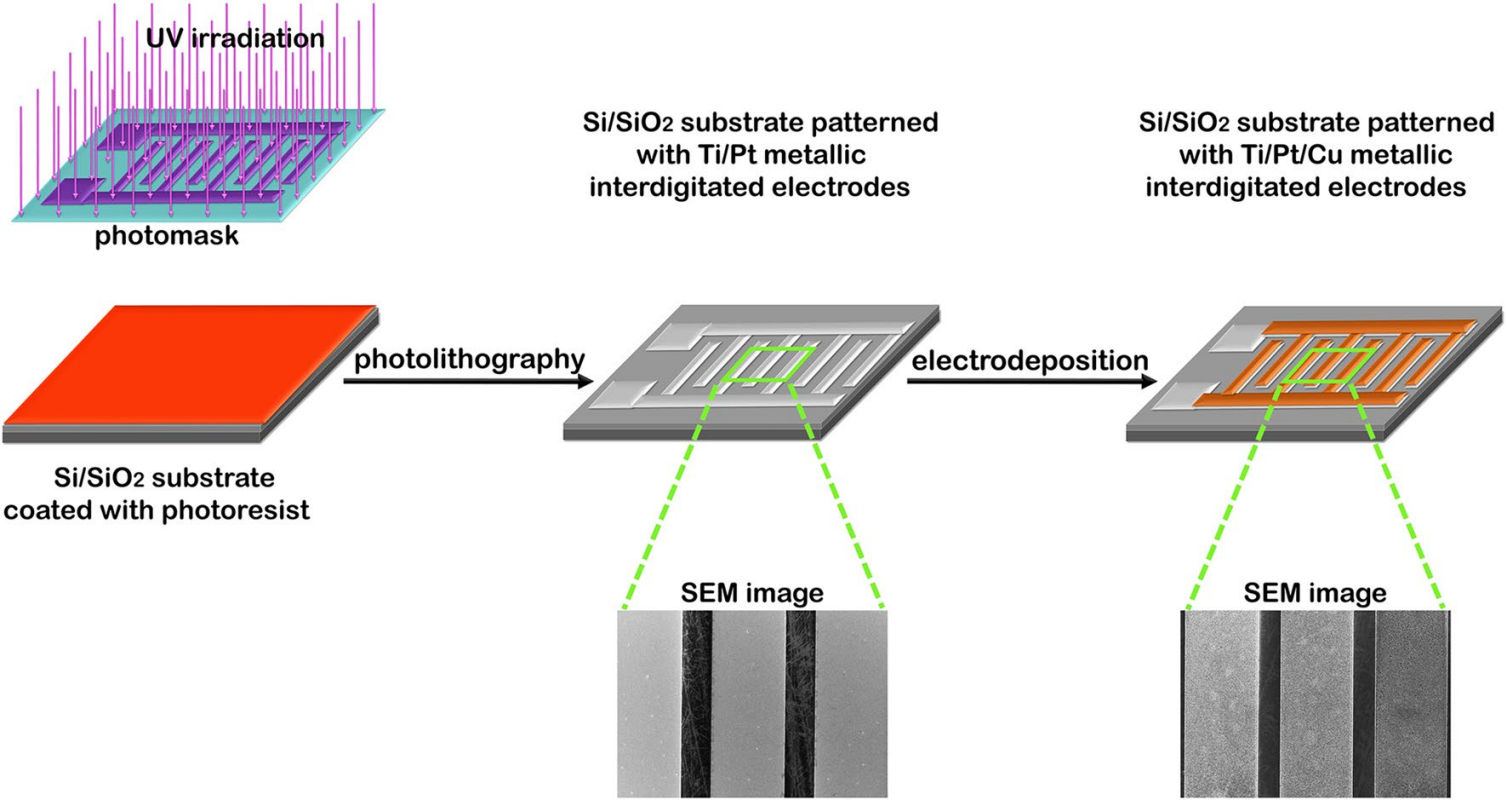
Schematic representation of the main steps implicated in the fabrication of the Ti/Pt/Cu metallic interdigitated electrodes on Si/SiO_2_ substrates.

A schematic representation with the main steps implicated in the fabrication of the Ti/Pt/Cu metallic interdigitated electrodes on Si/SiO_2_ substrates is sketched in Fig. 1.

In the first step, a conventional photolithographic process was employed to obtain Si/SiO_2_ wafers patterned with Ti/Pt metallic interdigitated electrodes, similar to our previously articles^47^, using a different geometry for the interdigitated electrodes, having 20 µm gaps between the electrodes (Supplementary Fig. S1). The photolithography process was made using an EVG 620 Mask Alignment System located into an ISO 5 cleanroom facility and a TECTRA RF magnetron sputtering equipment. The Si/SiO_2_ wafers (SiO_2_ thickness of ≈ 50 nm) were firstly cleaned in order to remove the contaminants. Further, an UV sensitive resist bought from MicroChemicals GmbH was spin coated on the surface of the Si/SiO_2_ wafers. Afterwards, the wafer coated with a thin layer of photoresist is thermally treated on a hotplate and then UV irradiated through a photomask. The resist covered Si/SiO_2_ wafers are developed using a developer acquired from MicroChemicals GmbH. After this process, a thin film of Ti (thicknesses of ≈ 10 nm) and Pt (thicknesses of ≈ 100 nm) were deposited by RF magnetron sputtering on the surface of the developed Si/SiO_2_ wafers. Subsequently, the remained photoresist on the Si/SiO_2_ wafers was stripped-off using a lift-off process in order to obtain Si/SiO_2_ wafers patterned with Ti/Pt metallic interdigitated electrodes.

In the second step, Ti/Pt/Cu metallic interdigitated electrodes are fabricated on Si/SiO_2_ substrates (Supplementary Fig. S2) by electrodepositing a Cu film on top of the Ti/Pt metallic interdigitated electrodes. First, the Si/SiO_2_ wafers patterned with Ti/Pt metallic interdigitated electrodes are diced (1 cm × 2 cm) in order to obtain single Si/SiO_2_ substrates patterned with Ti/Pt metallic interdigitated electrodes that are used further as working electrodes in the electrochemical deposition process of the Cu film. The deposition bath contains 220 g/l Cu_2_SO_4_·5H_2_O and 32 g/l H_2_SO_4_. The electrodeposition process was performed using a VoltaLab PGZ100 potentiostat, in a two-electrode configuration, consisting in a copper foil (2 cm × 2 cm × 2 mm) as anode and the Pt thin film of the Ti/Pt metallic interdigitated electrodes as the working electrode (cathode).

### Fabrication of the photodetector system based on self-connected CuO–ZnO core–shell heterojunction nanowire arrays grown on Ti/Pt/Cu metallic interdigitated electrodes

**Figure 2:**
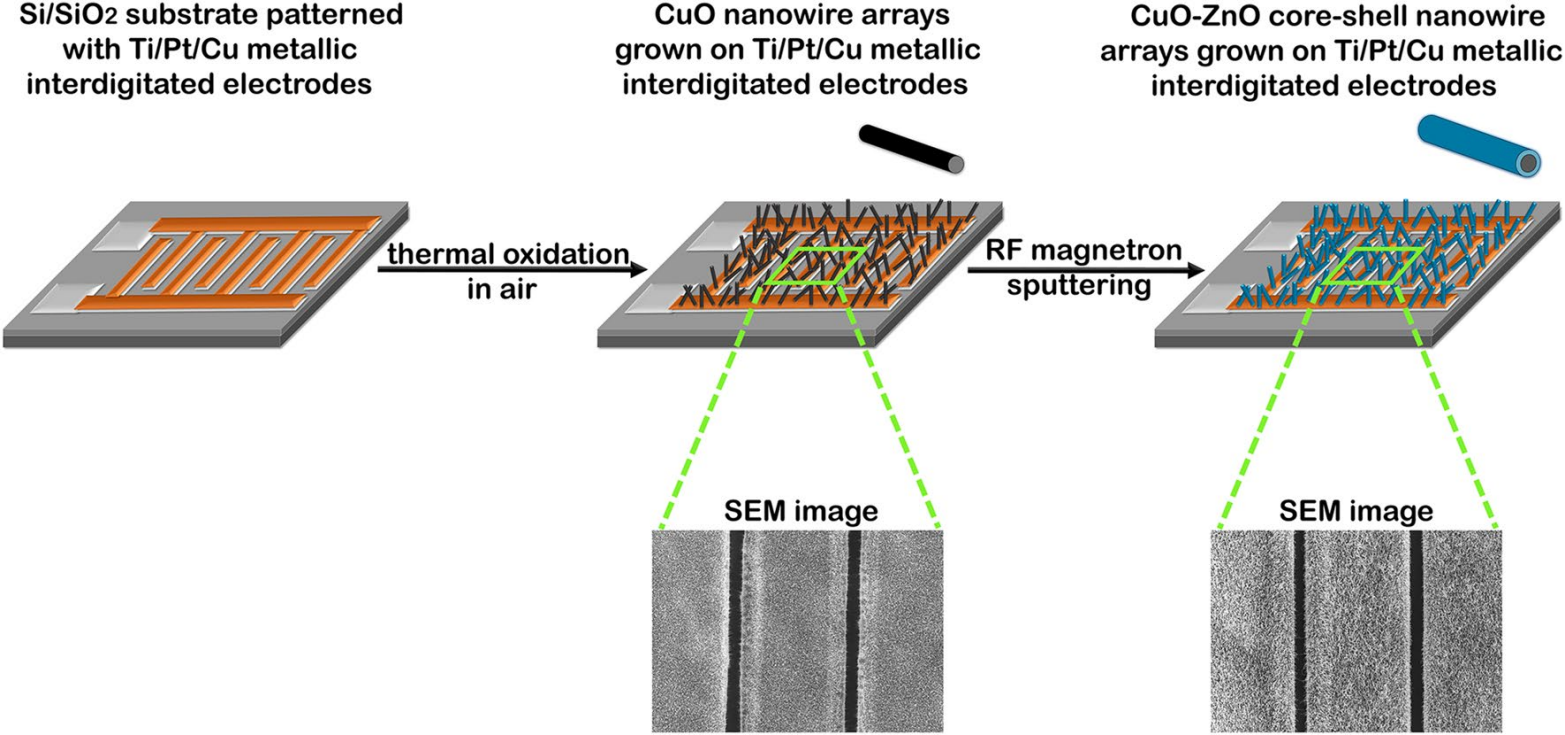
Schematic representation of the main steps involved in the development of the photodetector system based on self-connected CuO–ZnO core–shell heterojunction nanowire arrays grown on metallic interdigitated electrodes.

The main stages in the process of fabricating the photodetector system based on metallic interdigitated electrodes with self-connected CuO–ZnO core–shell heterojunction nanowire arrays are sketched in Fig. 2.

The first step is to grow CuO nanowire arrays on top of the metallic interdigitated electrodes. The top copper layer of the electrode system is thermally oxidized in air, in a simple and effective approach by applying similar experimental parameters as in our previous work^28^. Thus, the diced Si/SiO_2_ substrates patterned with Ti/Pt/Cu metallic interdigitated electrodes were placed on a ceramic substrate and introduced into a convection oven (Nabertherm GmbH) were the Cu film is thermally oxidized at 450 °C for 12 h in the ambient pressure. The second step is focused on the preparation of the CuO–ZnO core–shell nanowire arrays on top of the metallic interdigitated electrodes. Thus, the surface of the as prepared CuO nanowire arrays was uniformly covered by a thin film of ZnO by employing radio-frequency (RF) magnetron sputtering (Tectra GmbH Physikalische Instrumente). The following parameters were applied in the deposition process: 5.4 × 10^–3^ mbar pressure in the sputtering chamber, 100 W RF power applied on the magnetron and Ar atmosphere as the working gas. Two deposition times, 20 and 30 min, were used in order to investigate the influence of the ZnO shell thickness on the photoelectrical properties of the fabricated devices. The investigated samples were labelled as follows: CuO_MIE (self-connected CuO nanowire arrays grown on metallic interdigitated electrodes), CuO-ZnO_MIE_1 (self-connected CuO–ZnO core–shell nanowire arrays grown on metallic interdigitated electrodes with 20 min ZnO deposition time) and CuO-ZnO_MIE_2 (self-connected CuO–ZnO core–shell nanowire arrays grown on metallic interdigitated electrodes with 30 min ZnO deposition time).

### Morphological, structural and optical characterization

The morphology and the crystalline structure of the Ti/Pt metallic interdigitated electrodes with self-connected CuO nanowire and CuO–ZnO nanowire arrays were investigated using a Zeiss Merlin Compact field emission scanning electron microscope (FESEM) and a Bruker AXS D8 Advance instrument with Cu Ka radiation, λ = 0.154 nm X-ray diffractometer (XRD). The elemental composition of the metallic interdigitated electrodes was assessed by energy dispersive X-ray spectroscopy (EDX) in SEM using a Zeiss Evo 50 XVP scanning electron microscope (SEM) with an energy dispersive X-ray analysis (EDX) Quantax Bruker 200 as accessory The morphology, atomic structure and local chemical composition of the CuO and CuO–ZnO nanowires were evaluated with a high-resolution transmission electron microscope Cs probe-corrected JEM ARM 200F analytical electron microscope (TEM) including EDX elemental mapping in scanning transmission electron microscopy (STEM) and selected area electron diffraction (SAED). The optical reflectance spectra of the Ti/Pt metallic interdigitated electrodes with self-connected CuO nanowire and CuO–ZnO nanowire arrays were recorded with a Perkin–Elmer Lambda 45 UV–Vis spectrophotometer equipped with an integrating sphere.

### Electrical and photoresponse measurements

The current–voltage characteristics, in dark and under illumination, of the photodetector system based on metallic interdigitated electrodes with self-connected CuO–ZnO core–shell heterojunction nanowire arrays were measured in the ambient pressure and temperature using a Keithley 2400 Source Meter and a Oriel VeraSol-2 Class AAA LED Solar Simulator which has 19 independently controlled LED wavelengths from 400 to 1100 nm. The solar simulator was calibrated with an NREL certified KG5 filtered Si reference diode to an irradiation intensity of 100 mW/cm^2^.

## Results and discussion

### Morphological, structural and optical properties

**Figure 3:**
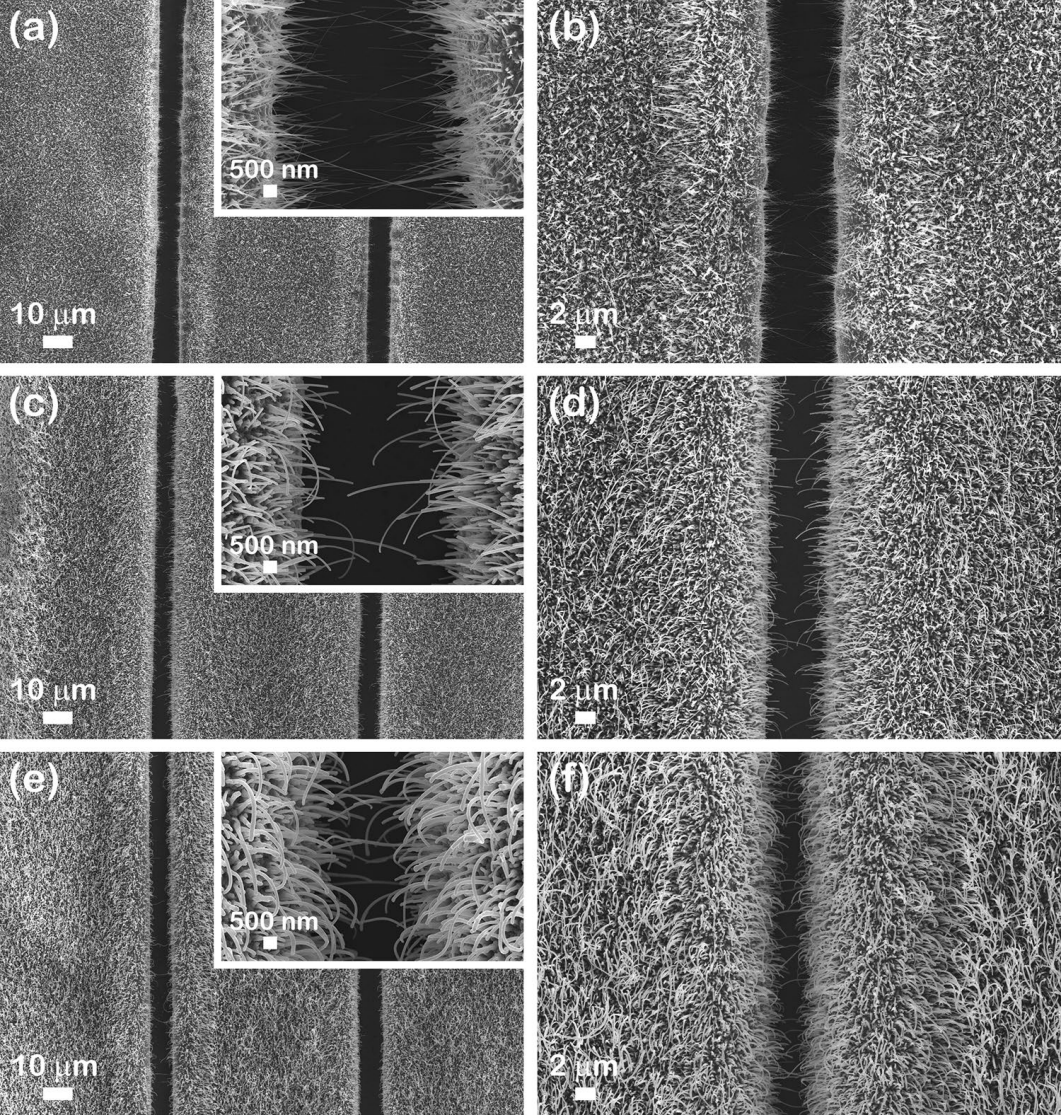
FESEM images at different magnifications of: (**a**,**b**) Inset CuO_MIE, (**c**,**d**) Inset CuO–ZnO_MIE_1 and (**e**,**f**) Inset CuO–ZnO_MIE_2.

The morphological properties of the CuO_MIE, CuO–ZnO_MIE_1 and CuO–ZnO_MIE_2 samples were investigated by FESEM, the results being illustrated in Fig. 3.

At lower magnification, the FESEM images of the samples show that the metallic interdigitated electrodes are uniformly covered by CuO nanowire arrays (Fig. 3a,b) or by CuO–ZnO core–shell nanowire arrays (Fig. 3c–f). Regardless their chemical composition, the nanowires are cylindrical in shape and have a high aspect ratio. Furthermore, the nanowires are vertically grown on the metallic interdigitated electrodes, at the electrodes edges, the nanowires being grown tilted or even horizontal. The FESEM images at a higher magnification evidenced that the CuO nanowires (Fig. 3a Inset) and the CuO–ZnO core–shell nanowires (Fig. 3c Inset and e Inset) are self-connected between the gaps of the metallic interdigitated electrodes.

**Figure 4:**
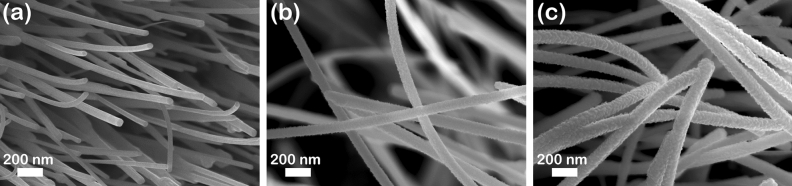
FESEM images at higher magnification of: (**a**) CuO_MIE, (**b**) CuO–ZnO_MIE_1 and (**c**) CuO–ZnO_MIE_2.

Additionally, it can be noticed that the RF magnetron sputtering process had as a result a uniform and conformal coverage (without any agglomerations or irregularities) of the pristine CuO nanowires with the ZnO thin film shell (Fig. 4). Hence, all type of nanowires have a smooth surface with lengths up to 10 µm and an average diameter of about 50 nm for the pristine CuO nanowires (Fig. 4a), 80 nm for the CuO–ZnO_MIE_1 core–shell nanowires (Fig. 4b) and 90 nm for the CuO–ZnO_MIE_2 core–shell nanowires (Fig. 4c). Consequently, the ZnO thin film shell thickness was estimated to be about 15 nm for CuO–ZnO_MIE_1 sample and 20 nm for CuO–ZnO_MIE_2 sample.

**Figure 5:**
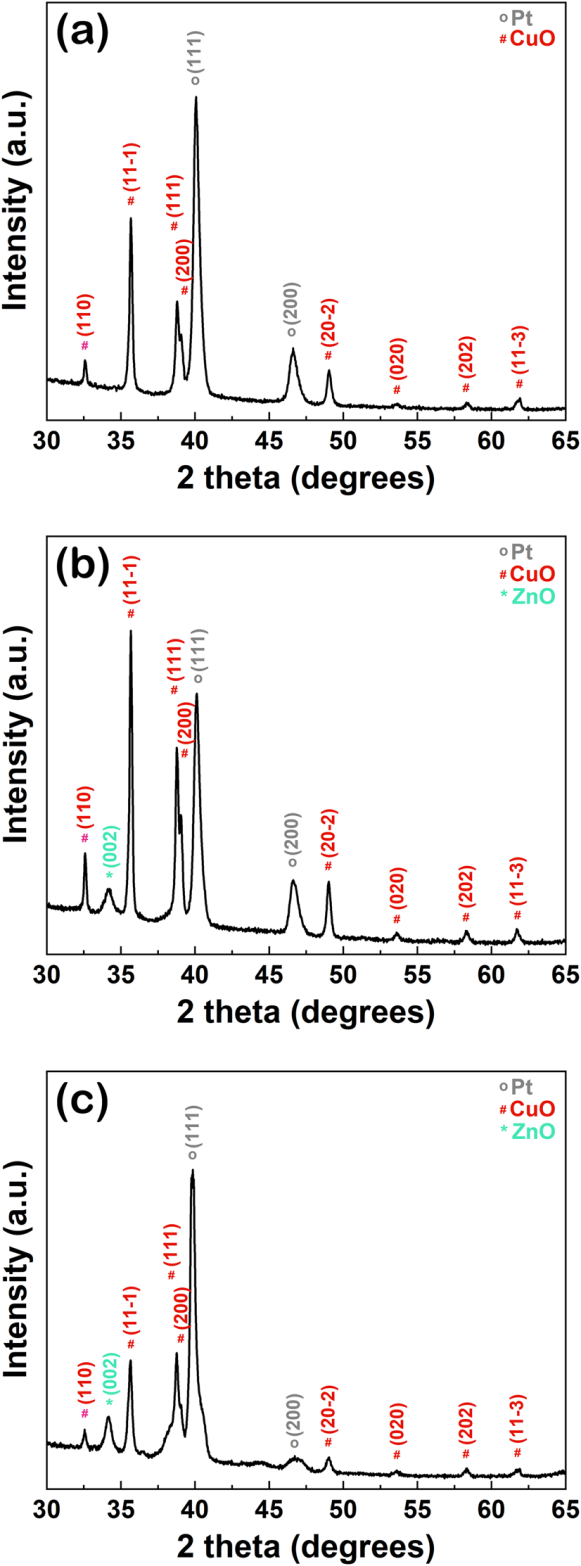
XRD patterns of: (**a**) CuO_MIE, (**b**) CuO–ZnO_MIE_1 and (**c**) CuO–ZnO_MIE_2.

**Figure 6:**
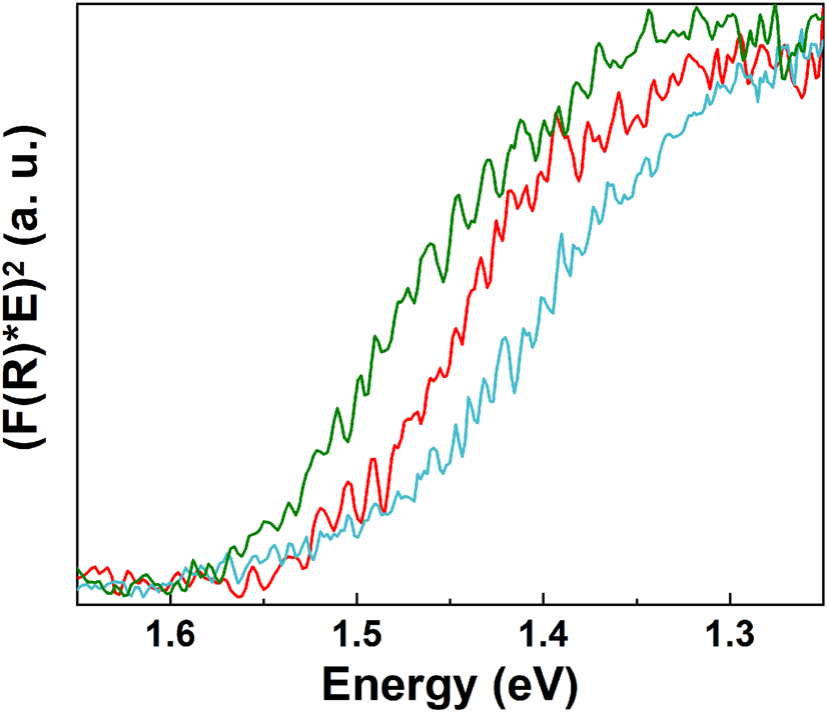
Kubelka–Munk function representations for: CuO_MIE (red curve), CuO–ZnO_MIE_1 (cyan curve) and CuO–ZnO_MIE_2 (olive curve).

The structural and optical properties were evaluated directly on the as obtained self-connected nanowire arrays grown on metallic interdigitated electrodes, being presented in Figs. 5 and 6, respectively. Thus, in the XRD pattern for the CuO_MIE sample (Fig. 5a), the diffraction peaks from 2θ = 39.7° and 46.2° are related to the Miller indexes of the (111) and (200) reflecting planes for Pt in cubic phase: (JCPDS reference code 00-004-0802), while the diffraction peaks from 2θ = 32.5°, 35.5°, 38.7°, 38.9°, 48.7°, 53.4°, 58.2° and 61.5° are linked to the Miller indexes of the (110), (11-1), (111), (200), (20-2), (020), (202) and (11-3) reflecting planes for CuO in monoclinic phase (JCPDS reference code 00-048-1548). The XRD patterns recorded for the CuO–ZnO core–shell nanowire samples (CuO–ZnO_MIE_1 and CuO–ZnO_MIE_2), illustrated in Fig. 5b,c, revealed besides the diffraction maxima attributed to Pt metallic layer and CuO nanowires (core), an additionally peak at 2θ = 34.4° assigned to the Miller indexes of the (002) reflecting plane for ZnO in a hexagonal wurtzite phase (JCPDS reference code 00-036-1451), confirming the deposition of the ZnO shell thin film.

The band gap of semiconductor materials represent a key feature that strongly influence their application in optoelectronic devices. Therefore, the optical properties of the samples were evaluated by diffuse reflectance spectroscopy in order to determine the band gap value for the nanowires, being evaluated based on the reflectance data (Supplementary Fig. S3) by Kubelka–Munk representation (Fig. 6). Hence, by plotting (F(R)*E)^2^ versus the photon energy (E) for all the samples: CuO_MIE (Fig. 6 red curve), CuO–ZnO_MIE_1 (Fig. 6 cyan curve) and CuO–ZnO_MIE_2 (Fig. 6 red curve), their band gap values where estimated to be in the range of 1.5–1.6 eV. F(R) is the Kubelka–Munk function, with F(R) = (1 − R)^2^/2R and R represent the measured diffuse reflectance. The estimated band gap values for the nanowires are in agreement with previously reported data for CuO nanowires^48^ and CuO–ZnO core–shell nanowires^28^.

**Figure 7:**
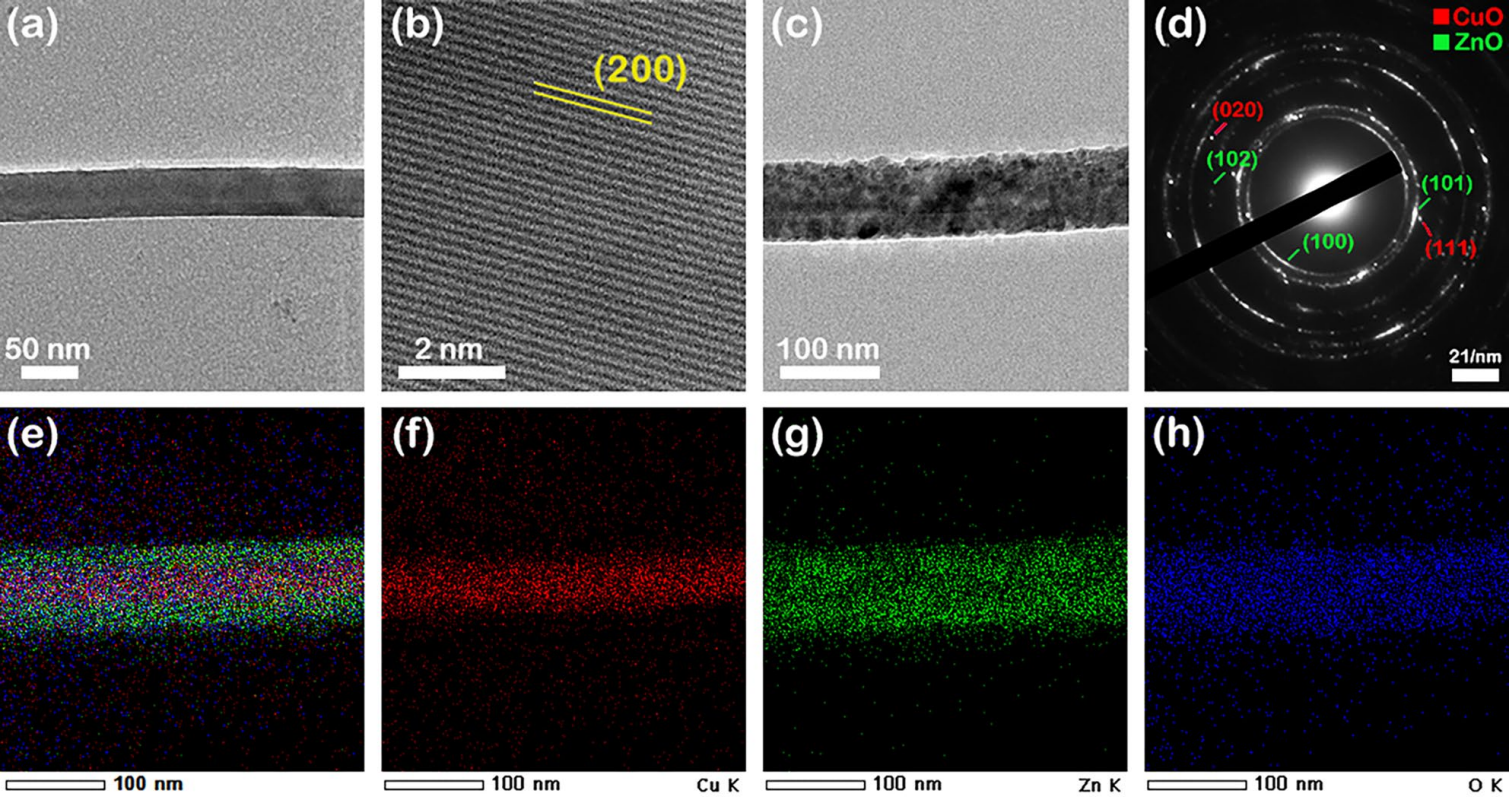
(**a**) TEM image and (**b**) HRTEM image of a single CuO nanowire (CuO_MIE sample), (**c**) TEM image and (**d**) SAED pattern of a single CuO–ZnO core–shell nanowire (CuO–ZnO_MIE_2 sample), (**e**–**h**) EDX elemental mapping of a single CuO–ZnO core–shell nanowire (CuO–ZnO_MIE_2 sample) and of the Cu, Zn and O as its individual constituent elements.

To prove the formation and to study the crystallographic structure of the CuO–ZnO core–shell heterojunction, TEM measurements, selected area electron diffraction (SAED), high-resolution transmission electron microscopy (HRTEM) and EDX mapping in STEM were carried out on the pristine CuO nanowires and CuO–ZnO core–shell nanowires. In order to perform the TEM investigations CuO nanowires and CuO–ZnO core–shell nanowires were harvested from the Ti/Pt/Cu metallic interdigitated electrodes by ultrasonication in ultrapure isopropyl alcohol to obtain a suspension of nanowires. The nanowires were placed on TEM grids by directly drop casting droplets of nanowires suspensions onto their surface. Thus, the TEM image of a single CuO nanowire (Fig. 7a) exhibits that the nanowire has a diameter of 50 nm, a smooth surface without defects and a cylindrical shape in accordance with the FESEM images (Figs. 3a inset, 4a). The HRTEM image of the selected area from the CuO nanowire (Fig. 7b) shows that the nanowire grows along the (200) direction of monoclinic structure with an interplanar spacing of d_(200)_ = 0.23 nm. The TEM image of a single CuO–ZnO nanowire (Fig. 7c) evidenced that the ZnO thin film shell uniformly covers the CuO nanowire core, the CuO–ZnO nanowire having a diameter of about 90 nm with a ZnO shell thickness estimated at about 20 nm, in agreement with the FESEM data (Figs. 3c inset, 4c). The SAED pattern (Fig. 7d) confirms that both CuO and ZnO heterojunction components have ring patterns corresponding to a polycrystalline structure, monoclinic (CuO core) and hexagonal wurtzite (ZnO shell) according to the XRD data (Fig. 5). Moreover, Fig. 7e–h display the EDX elemental mappings of a single CuO–ZnO core–shell nanowire and of the Cu K, Zn K and O K elements revealing the elemental spatial distribution of these elements across the entire volume of the CuO–ZnO core–shell nanowire. Hence, the Cu K is confined only in the core area, the Zn K being present more to the nanowire surface while the O K being evenly distributed along the entire volume of the CuO–ZnO core–shell nanowire.

### Optoelectronic properties

**Figure 8:**
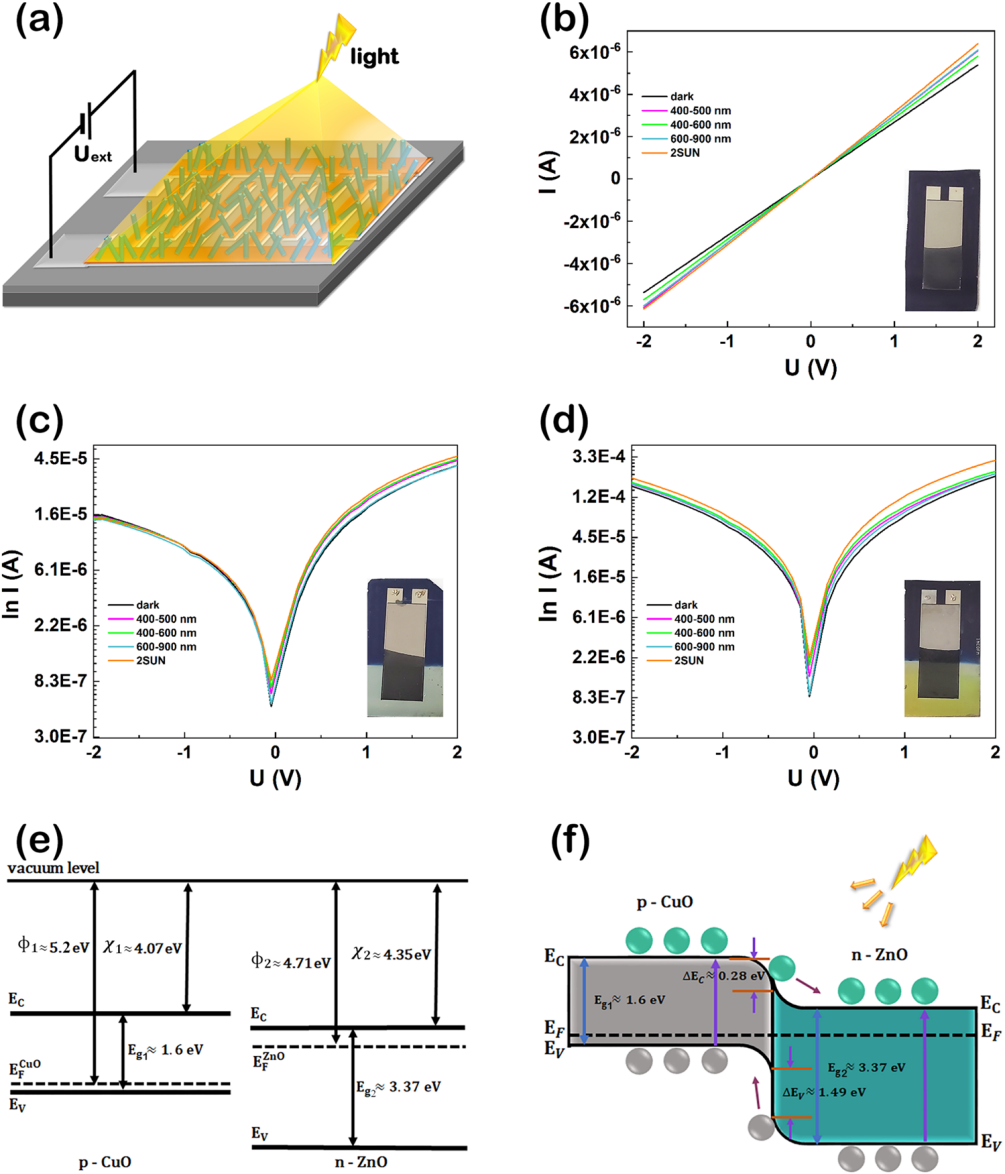
(**a**) Schematic representation of the photodetector system based on self-connected CuO–ZnO core–shell heterojunction nanowire arrays under illumination, (**b**) Current–voltage characteristic of CuO_MIE, I–U semilogarithmic plot of the photodetector systems: (**c**) CuO–ZnO_MIE_1 and (**d**) CuO–ZnO_MIE_2 under dark (black) and under illumination with different spectral intervals: 400–500 nm (magenta), 400–600 nm (green), 600–900 nm (cyan) and 2 SUN (orange), Schematic representations of the band diagrams for: (**e**) CuO and ZnO and (**f**) CuO–ZnO core–shell heterojunction. Insets: (**b–d**) Optical images of the investigated samples.

A major advantage of the photodetector system developed in the current study consists in the fact that the electrical connections are achieved without any additional complex and time-consuming lithographic contacting step due to the self-connected CuO–ZnO core–shell heterojunction nanowire arrays grown directly onto the Ti/Pt metallic interdigitated electrodes. A schematic representation with the fabricated photodetector is depicted in Fig. 8a. The electrical measurements of the samples were carried out at room temperature and atmospheric pressure in a conventional 2-points configuration in dark and under illumination with different spectral intervals using a Solar simulator.

The current–voltage characteristics of the self-connected CuO nanowire arrays grown on metallic interdigitated electrodes in dark conditions and under illumination with different spectral intervals (400–500 nm—magenta, 400–600 nm—green, 600–900 nm—cyan and 2 SUN—orange) are presented in Fig. 8b. Regardless of the conditions used, a linear shape is evidenced, indicating an Ohmic behaviour with a current of 1.42 µA in dark and 1.67 µA under illumination with 2 SUN at 0.5 V applied, with only a small photocurrent gain. Figure 8c,d reveal the semilogarithmic plot of the current–voltage characteristics of the two photodetectors based on self-connected CuO–ZnO core–shell heterojunction nanowire arrays grown on metallic interdigitated electrodes (CuO–ZnO_MIE_1 and CuO–ZnO_MIE_2) in dark and under illumination with different spectral intervals (400–500 nm—magenta, 400–600 nm—green, 600–900 nm—cyan and 2 SUN—orange). For both types of photodetectors, it can be noticed a non-linear shape, emphasizing a rectifying behaviour that originate from the p–n radial heterojunction formed at the CuO/ZnO interface in the CuO–ZnO core–shell nanowires. Additionally, it can be observed that the current flow increases with the increase of the ZnO shell thickness, exhibiting a current of 11.41 µA for CuO–ZnO_MIE_1 and 38.23 µA for CuO–ZnO_MIE_2 under illumination with 2 SUN at 0.5 V applied bias. The result can be explained taking into account the difference in the ZnO shell thickness of the two samples: ~ 15 nm for CuO–ZnO_MIE_1 and ~ 20 nm for CuO–ZnO_MIE_2. The schematic representation of the p-type CuO and n-type ZnO band diagrams is given in Fig. 8e. Hence, the bandgaps of CuO and ZnO at room temperature are ${E}_{g1}\approx 1.6 \mathrm{eV}$ and ${E}_{g2}\approx 3.37 \mathrm{eV}$, respectively^48,49^. In addition, the electron affinities and work functions of CuO and ZnO are ${\chi }_{1}\approx 4.07 \mathrm{eV}$, ${\phi}_{1}\approx 5.2 \mathrm{eV}$ and ${\chi }_{2}\approx 4.35 \mathrm{eV}$, ${\phi}_{2}\approx 4.71 \mathrm{eV}$ respectively^50–52^. The schematic representation of the band diagram after joining the two semiconductor materials into a radial CuO–ZnO core–shell heterojunction in the nanowires under illumination is depicted in Fig. 8f.

Taking into account the values of the bandgaps and electron affinities of the CuO and ZnO, the conduction band offset (${\Delta E}_{C}={\chi }_{2}-{\chi }_{1}$) and the valence band offset (${\Delta E}_{V}={(E}_{g2}-{E}_{g1})-{\Delta E}_{C}$) of the CuO–ZnO core–shell heterojunction were ${\Delta E}_{C}=0.28 \mathrm{eV}$ and ${\Delta E}_{V}=1.49 \mathrm{eV}$ respectively. The radial p–n heterojunction formed between CuO and ZnO generates a depletion layer with staggered gap type II band alignment that favors an efficient charge separation of the photogenerated electron–hole pairs at the CuO–ZnO interface, suppressing their recombination and consequently enhancing the resulting photocurrent. Thus, under light illumination of the CuO–ZnO core–shell heterojunction, the electrons from the valence band (${E}_{V}$) of the CuO and ZnO are excited in the conduction band (${E}_{C}$), holes are generated in the valence band resulting electron–hole pairs. Then, the photogenerated electrons from the CuO conduction band migrate into the ZnO conduction band, whereas the photogenerated holes from the ZnO valence band migrate towards the CuO valence band. Accordingly, the photogenerated charges are suppressed to recombine leading to an increase of the charge collection efficiency at the interface and to the enhancement of the photocurrent and responsivity of the photodetector device. The formation of the type II band alignment between CuO and ZnO in the core–shell nanowires is proved by the increase in the photocurrent for the photodetectors based on self-connected CuO–ZnO core–shell heterojunction nanowire arrays grown on metallic interdigitated electrodes (Fig. 8c,d), comparing with the device based only on CuO nanowire arrays (Fig. 8b).

**Figure 9:**
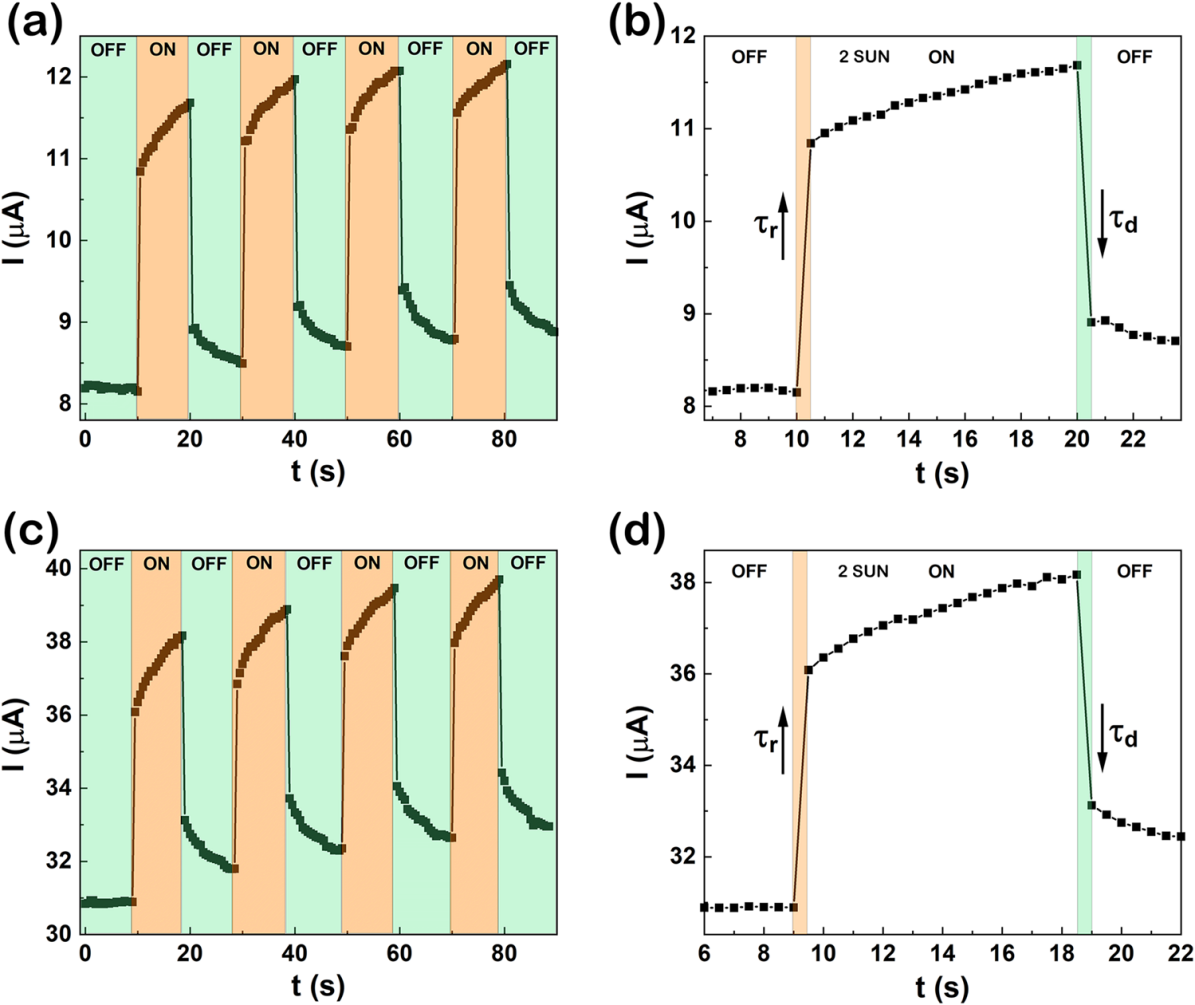
(**a**,**c**) Time-dependent photoresponse and (**b**,**d**) rise and decay time at 0.5 V applied bias during the light ON/OFF cycles on the photodetector systems: (**a**,**b**) CuO–ZnO_MIE_1 and (**c**,**d**) CuO–ZnO_MIE_2.

The time-dependent photoresponse measurements were recorded under 2 SUN illumination with light ON/OFF cycles at 0.5 V applied bias on the photodetectors (CuO–ZnO_MIE_1 and CuO–ZnO_MIE_2) being evidenced in Fig. 9. From the time-dependent photoresponse (Fig. 9a,c), calculating the difference between photocurrent and dark current (${I}_{light}/{I}_{dark}$) for each type of photodetector, it can be noticed that the net current is about 3 µA for CuO–ZnO_MIE_1 and 6.5 µA for CuO–ZnO_MIE_2 under illumination with 2 SUN at 0.5 V applied bias. Moreover, the photodetectors are stable in time, taking into account that after four ON/OFF switching cycles, it was observed that the ratio between photocurrent and dark current is approximately the same even if there is an increase of the photocurrent in time under illumination for both type of devices. The increase of the photocurrent in time can be due to the continuous illumination that can increase the temperature, lowering the resistivity and increasing the current^28^, or can be linked to the decrease of the photoinduced surface depletion depth^35^. The rise time (τ_r_) and decay time (τ_d_) of the photodetectors were estimated based on the enlarged time-dependent photoresponse (Fig. 9b,d). The obtained values are τr = 0.5 s and τd = 0.5 s for both type of photodetectors CuO–ZnO_MIE_1 and CuO–ZnO_MIE_2.

The performances of the photodetectors can be assessed by determining the most important figures of merit of the optoelectronic devices: responsivity (R), specific detectivity (D*) and external quantum efficiency (EQE). Responsivity represents the generated photocurrent per unit of the incident light power on the effective illuminated area of a photodetector. Detectivity is a key parameter that characterizes the ability of a photodetector to detect weak signals from noise.

The figures of merit of a photodetector can be estimated using the following equations^53,54^:

1 $$R=\frac{{I}_{light}-{I}_{dark}}{PS},$$

2 $${D}^{*}=\frac{R}{\sqrt{\frac{2e{I}_{dark}}{S}}},$$

3 $$EQE=R\frac{hc}{e\lambda },$$

where ${I}_{light}$ is the photocurrent, ${I}_{dark}$ is the dark current, P is the incident illumination power, S is the effective illuminated area, e is the elementary charge, λ is the light wavelength, c is the speed of light, e is the elementary charge and h is the Planck constant.

Taking into account that the effective illuminated area is 0.49 mm^2^ for each sample and the above equations, the figures of merit of the two type of photodetectors were estimated to be: 12.3 A/W and 5.34 × 10^13^ Jones for CuO–ZnO_MIE_1 and 26.3 A/W and 5.85 × 10^13^ Jones for CuO–ZnO_MIE_2, respectively. Additionally, the external quantum efficiency parameter was calculated for the 400 nm wavelength, being 13.34% for CuO–ZnO_MIE_1 and 38.75% for CuO–ZnO_MIE_2, respectively. The performances of the photodetectors fabricated in the current study were presented in a comparative manner with the performances of state-of-the-art core–shell nanowire arrays based photodetectors, being illustrated in Table 1. Comparing with other photodetectors based on core–shell nanowire arrays, it can be observed that our photodetectors have similar performances, suggesting that the CuO–ZnO radial core–shell heterojunction nanowire arrays based photodetectors from the current study are promising candidates as visible-light photodetectors. However, our devices have the major advantage of simple fabrication, the electrical connections of the proposed photodetector systems being made without any additional complex and time-consuming lithographic step due to the self-connected CuO–ZnO core–shell heterojunction nanowire arrays grown directly onto the Ti/Pt metallic interdigitated electrodes.

**Table 1:** Comparison of the performances of core–shell nanowire arrays based photodetectors.

Nanowires materials	Bias	Range of detection	Responsivity	Detectivity	Rise time/decay time	References
CuO–ZnO core–shell	0.5 V	VIS	26.3 A/W	5.8 × 10^13^ Jones	0.5 s/0.5 s	This work
ZnO/Cu_2_O core–shell	5 V	UV	26.9 A/W	6.3 × 10^9^ Jones	1.4 s/3.4 s	^55^
ZnO/Cu_2_O core–shell	0 V	VIS	7.6 µA/W	–	0.09 s/0.09 s	^56^
ZnO/Co_3_O_4_ core–shell	0.1 V	VIS	21.8 mA/W	4.1 × 10^12^ Jones	6 s/–	^35^
ZnO/Ga_2_O_3_ core–shell	0 V	UV	137.9 mA/W	6.1 × 10^12^ Jones	28.9 µs/85.7 µs	^53^
ZnO/ZnS core–shell	1.5 V	UV	0.2 A/W	–	–	^57^
ZnO/CuCrO_2_ core–shell	− 1 V	UV	5.87 mA/W	8.5 × 10^9^ Jones	32 µs/35 µs	^33^

## Conclusions

In summary, the present work reports a novel visible-light photodetector system based on self-connected CuO–ZnO radial core–shell heterojunction nanowire arrays grown on metallic interdigitated electrodes. It was demonstrated that the radial p–n heterojunction formed in the CuO–ZnO core–shell nanowires enhances the photoresponse and photoresponsivity of the photodetectors. The nanowire based photodetectors were fabricated using simple and cost-effective preparation methods, such as photolithography, electrodeposition, thermal oxidation in air and RF magnetron sputtering. The morphological properties evidence a diameter of 80 nm for the CuO–ZnO_MIE_1 core–shell nanowires and 90 nm for the CuO–ZnO_MIE_2 core–shell nanowires. The structural characterization revealed a monoclinic phase for the CuO nanowires and a hexagonal wurtzite phase for the ZnO thin film shell. The band gap values of the CuO–ZnO core–shell nanowire arrays were estimated in the range of 1.5–1.6 eV based on the reflectance data. The electrical and photoresponse measurements proved that the photodetectors based on self-connected CuO–ZnO radial core–shell heterojunction nanowire arrays grown on metallic interdigitated electrodes operate as visible-light photodetector with the following figures of merit: 12.3 A/W and 5.34 × 10^13^ Jones for CuO–ZnO_MIE_1 and 26.3 A/W and 5.85 × 10^13^ Jones for CuO–ZnO_MIE_2, respectively. Furthermore, the rise and decay times of the photodetectors were τr = 0.5 s and τd = 0.5 s. The major advantage of the fabricated photodetectors consists in the fact that the electrical connections are made in a straightforward manner (the self-connected CuO–ZnO core–shell heterojunction nanowire arrays were grown directly onto the Ti/Pt metallic interdigitated electrodes) avoiding complex and time-consuming lithographic steps. The current study highlights that such photodetectors based on self-connected CuO–ZnO core–shell heterojunction nanowire arrays can have a potential impact on the development of the future nanoscale optoelectronic devices.

## Supplementary Information

Supplementary Figures.

## Data Availability

The datasets supporting the conclusions of the current study are presented in the manuscript and supporting information.
