# Author Correction: Self-connected CuO–ZnO radial core–shell heterojunction nanowire arrays grown on interdigitated electrodes for visible-light photodetectors

**DOI:** 10.1038/s41598-022-15047-3

**Published:** 2022-06-23

**Authors:** Andreea Costas, Camelia Florica, Nicoleta Preda, Cristina Besleaga, Andrei Kuncser, Ionut Enculescu

**Affiliations:** National Institute of Materials Physics, Nanostructures Laboratory, 405A Atomistilor Street, 077125 Magurele, Ilfov Romania

Correction to: *Scientific Reports* 10.1038/s41598-022-10879-5, published online 27 April 2022

The Acknowledgements section in the original version of this Article was incomplete.

“This work has been funded by the Executive Agency for Higher Education, Research, Development and Innovation Funding (UEFISCDI), Romania, Project code: PN-III-P1-1.1-PD-2019-1102 and by the Core Program, contract no. PN19-03 (contract no. 21 N/08.02.2019) supported from the Romanian Ministry of Research and Innovation.”

now reads:

“This work has been funded by the Executive Agency for Higher Education, Research, Development and Innovation Funding (UEFISCDI), Romania, Project code: PN-III-P1-1.1-PD-2019-1102 and by the Core Program, contract no. PN19-03 (contract no. 21 N/08.02.2019) supported from the Romanian Ministry of Research and Innovation. The fee for open access publication was supported from the project 35PFE/2021, funded by the Romanian Ministry of Research, Innovation and Digitization.”

The original Article has been corrected.

