# Supplementary material for: Self-connected CuO–ZnO radial core–shell heterojunction nanowire arrays grown on interdigitated electrodes for visible-light photodetectors
Source: Sci Rep. 2022 Apr 27;12:6834. doi: 10.1038/s41598-022-10879-5 (PMC9046224; doi:10.1038/s41598-022-10879-5)
Supplement: Supplementary file 1 — Supplementary Figures. [file 41598_2022_10879_MOESM1_ESM.docx]

Self-connected CuO-ZnO radial core-shell heterojunction nanowire arrays grown on interdigitated electrodes for visible-light photodetectors

Andreea Costas^#*1^, Camelia Florica^#1^, Nicoleta Preda^1^, Cristina Besleaga^1^, Andrei Kuncser^1^ &

Ionut Enculescu^**1^

^1^National Institute of Materials Physics, Nanostructures Laboratory, 405A Atomistilor Street, 077125, Magurele, Ilfov, Romania

^#^Andreea Costas and Camelia Florica contributed equally to this work.

^*^Corresponding author: [andreea.costas@infim.ro](mailto:andreea.costas@infim.ro) (A. Costas)

^**^Corresponding author: [encu@infim.ro](mailto:encu@infim.ro) (I. Enculescu)


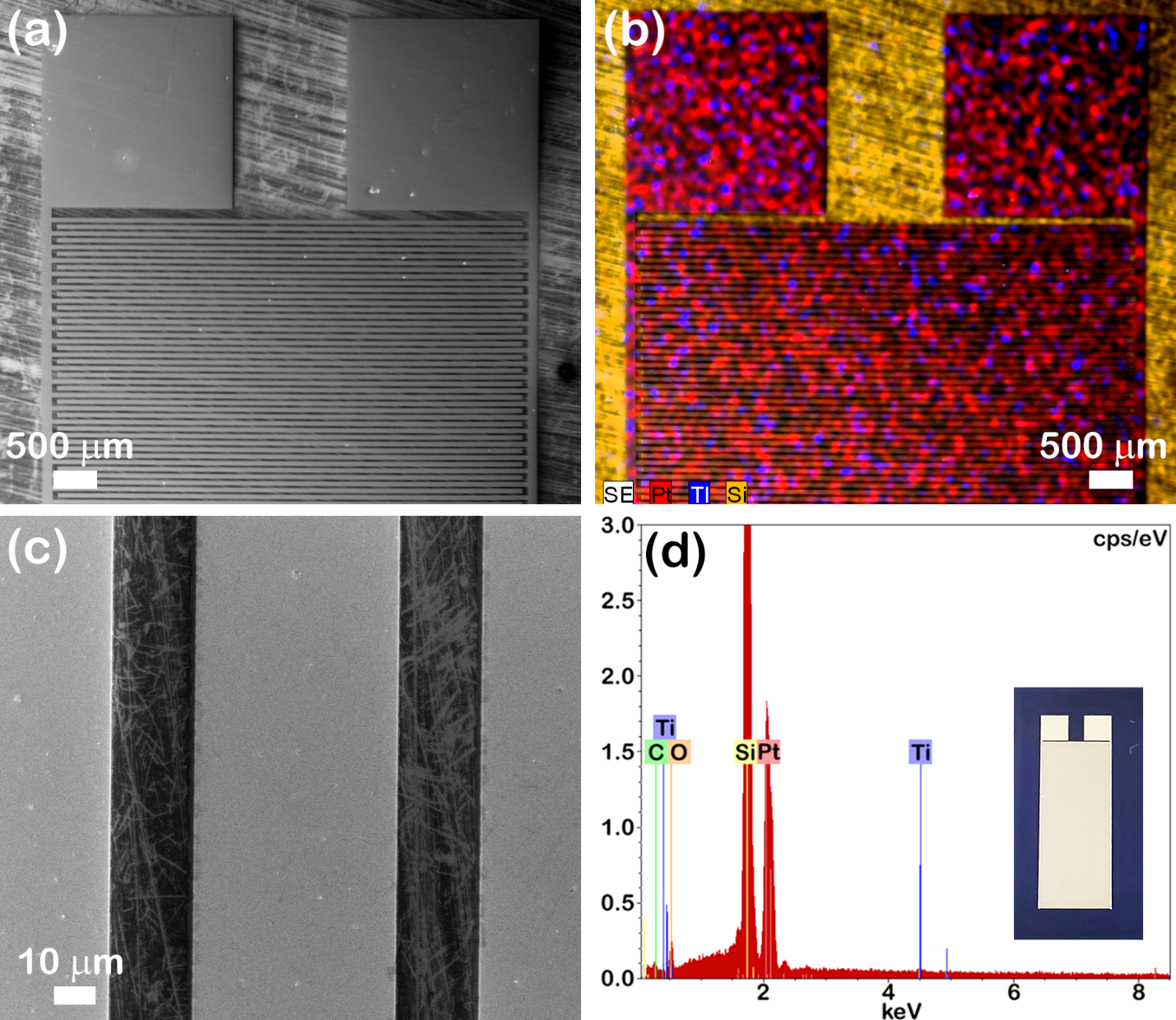


Figure S1. (a), (c) SEM images at different magnifications, (b) EDX mapping image and (d) EDX spectrum of the Ti/Pt metallic interdigitated electrodes fabricated on Si/SiO_2_ substrates.


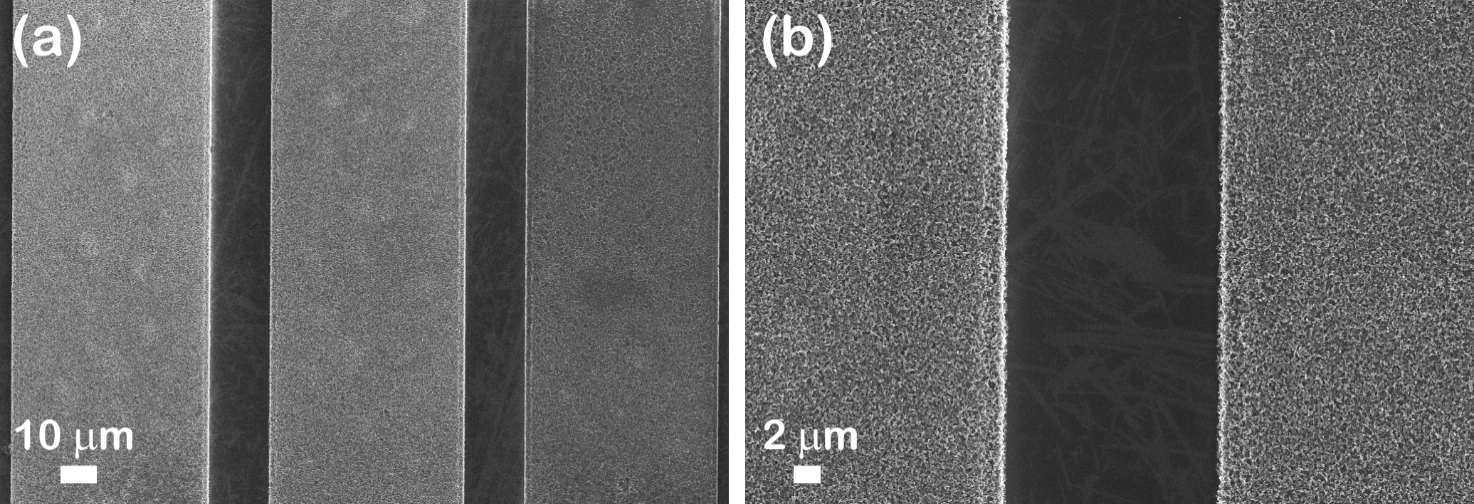


Figure S2. (a), (b) FESEM images at different magnifications of the Ti/Pt/Cu metallic interdigitated electrodes fabricated on Si/SiO_2_ substrates.





Figure S3. Reflectance spectra for: CuO_MIE (red curve), CuO-ZnO_MIE_1 (cyan curve) and CuO-ZnO_MIE_2 (olive curve).
